# Family Influences on Caries in Grenada

**DOI:** 10.3390/dj8030105

**Published:** 2020-09-09

**Authors:** Danielle Mitnick, Ashley Dills, Amy M. Smith Slep, Richard E. Heyman, Jill Giresi

**Affiliations:** Department of Cariology and Comprehensive Care, New York University College of Dentistry, New York, NY 10010, USA; Danielle.Mitnick@nyu.edu (D.M.); Amy.Slep@nyu.edu (A.M.S.S.); Richard.Heyman@nyu.edu (R.E.H.); Jill.Giresi@gmail.com (J.G.)

**Keywords:** family environment, early childhood caries, cultural competency, Grenada, parenting, discipline, motivational interviewing

## Abstract

If high-conflict family environments are cariogenic across cultures, and can be studied in cultures where both these environments and cariogenic dental practices are particularly prevalent, this would afford the opportunity to examine how these two pathways of risk might interact, laying the stage for culturally competent, integrated prevention efforts. The first investigation involved qualitative data about perceptions of oral health and family stressors in Grenadian families with school-aged children. The second study used a questionnaire and observational data to assess relations among oral health behaviors, relationship satisfaction, parenting, and child behavior; it also included a pilot study of Motivational Interviewing. Most of the themes discussed in focus groups suggested overlap between U.S. and Grenadian parents; possible culture-specific issues were high prevalence of single-parent homes, normativity of physical discipline, less preventive dental care, and more fatalistic view of oral health outcomes. Significant associations were found between parent and child oral health behaviors, between child externalizing and internalizing behaviors, and between family variables (e.g., relationship satisfaction, child behavior) and oral health behaviors (e.g., parent flossing, child brush time). The results strongly support the need for research on the relations between family functioning and oral health to be embedded within culture.

## 1. Introduction

### 1.1. Current Interventions for Early Childhood Caries

Early childhood caries (ECC) results in pain, higher risk for caries in permanent teeth, and compromised physical, social, and cognitive development [1]. Although the etiological influence of oral bacteria has been well-supported, preventive interventions for ECC that have targeted oral bacteria (i.e., that attempt to reduce cariogenic bacteria in at-risk populations via health education and dietary advice) result in small, short-term effects [2] and may not be generalizable outside the study [3]. Motivational Interviewing (MI; [4]), an approach that allows clients to explore problems according to their own stage of change, has accumulating evidence of effectiveness for reducing ECC [5], but it has not been tested cross-culturally.

High-conflict family environments may be a missing key target in the prevention of ECC. A recent U.S. study suggests that inter-parental emotional aggression is linked with adults’ and children’s caries [6]. Further examination has implicated children’s greater consumption of cariogenic beverages as a mechanism explaining this association [7]. However, children’s behavior is nested within family, community, and cultural ecologies [8]. It is uncertain whether family-based preventative interventions from high-income countries are portable to countries with considerably less access to preventive care, and where expectations regarding cariogenic child behavior, parental monitoring of cariogenic and/or preventive behavior, and family and community risk and protective factors differ. If high-conflict family environments are cariogenic across cultures, and can be studied in cultures where both they and cariogenic dental practices are particularly prevalent, this would afford the opportunity to examine how these two pathways of risk interact and lay the stage for well-informed, culturally competent, integrated prevention efforts.

### 1.2. Family Maltreatment Prevalence in Grenada

Family maltreatment is a cariogenic family environment factor [9] that is common and problematic in Grenada, an island in the Caribbean. Intimate partner violence (IPV) is a serious concern for the country [9,10]. The rates of physical IPV in Grenada are two times the global average [11]. Likewise, parental discipline often consists of physical punishment, such as spankings and beatings, to “teach parental expectations” [12]. Research suggests that permissive parenting styles are highly common throughout Caribbean countries [13].

### 1.3. Culturally Competent Practice in Grenada

To examine the interaction of these pathways of risk and the generalizability of interventions to reduce cariogenic bacteria in at-risk populations, we looked to Grenada. The results of a national survey of Grenadian oral health revealed that one in three children are missing a tooth due to dental decay, and the average child had an additional six surfaces that were decayed [14]. This is likely associated with multiple individual behaviors, family practices, shared cultural beliefs, and culturally normative practices. A qualitative study reported that many Grenadians believe health is more strongly associated with heredity than preventive health measures, and health-seeking behavior, such as lifestyle changes, should not occur until one cannot carry out his or her daily routine [15].

Cultural beliefs and practices may be shaped by difficulties with accessibility to preventive care and oral health treatment. In 2000, the dentist-to-population ratio in Grenada was one dentist per 7000 individuals; in 2007, the ratio declined, at 1.1 dentists to 10,000 individuals [16,17]. This is in contrast to an average dentist-patient ratio of 7.1 to 10,000 individuals in European Union countries [18]. Further, dental healthcare in Grenada is costly [14].

The current paper reports on two studies designed to understand how Grenadian families socialize and monitor oral health-related activities and family dynamics more broadly. The first study employed a focus group methodology to obtain qualitative data about the perceptions of oral health and family stressors in Grenadian families with school-aged children. We hypothesized that the themes of the focus groups would highlight similarities between Grenadian and U.S. families. The second study used questionnaire and observational data to empirically assess the relations among oral health behaviors, relationship satisfaction, parenting, and child behavior. We hypothesized that better functioning families would have better oral health behaviors.

## 2. Materials and Methods

Both studies were reviewed and approved in advance of recruitment by both the New York University’s Institutional Review Board (#13-9506) and St. George’s University’s (Grenada) Institutional Review Board.

### 2.1. Study 1: Procedure

Five focus group sessions were conducted with *N* = 50 Grenadian parents. Participants were recruited through fliers distributed at local primary schools. To meet eligibility, participants needed to be a parent of a child at the primary school and speak English. Participants were provided with study information, including the goal and purpose of the study, benefits and risks of participating, confidentiality, compensation, and contact information of study investigators, and informed consent was obtained. Facilitators used a structured set of questions to guide discussions (see Appendix A); no demographics were collected. Participants were compensated $25 US dollars, which is accepted in Grenada. Facilitators took detailed notes. Inductive thematic analysis was performed. All focus group content was coded by one of the authors, with a second author confirming the coding. Themes were discussed among all authors and facilitators.

### 2.2. Study 2: Procedure

Participants were *N =* 54 parents (48 mothers and 6 fathers) and their primary-school-aged children (ages 3.06–15.41 years old) recruited through fliers distributed at the same primary schools as in Study 1. To meet eligibility, participants needed to be part of the school community and speak English. Participants were invited to the Kalinago Beach Resort Hotel to complete questionnaires and have video-recorded conversations with their children. Transportation to and from the hotel was arranged, and participants were paid $25 US Dollars for their time.

Potential participants gave informed consent for themselves and assent for their children. Parents spent approximately one hour completing questionnaires. The questions were read out loud for participants who requested assistance. Demographic information is presented in Table 1).

Each parent-child dyad then spent 20 min participating in a video-recorded discussion task that has been employed in several studies with children in this age range [19]. The parent and child sat in a private area and were asked to work together for 10 min to plan a fun activity, such as going to the beach. Then, the parent and child problem-solved two issues, one problem that the mother wanted to discuss and one problem that the child wanted to discuss, which also lasted for a total of 10 min.

Finally, trained coaches conducted Motivational Interviews regarding oral health. The structure of the interviews was adapted from Weinstein et al., [20] and included establishing rapport, discussing parents’ own dental histories, discussing the parents’ concerns regarding their children’s oral health, presenting a menu of options to improve oral health, and allowing the parents to select any options they would like to try.

### 2.3. Study 2: Measures

Couple Satisfaction. The Couple Satisfaction Index (CSI-4) assesses intimate relationship satisfaction and has excellent convergent validity with other measures of relationship satisfaction [21]. Total scores range from 0–21, with a distress cutoff score of 13.5. Internal consistency in this study was α = 0.88.

Parental Discipline. The 11-item Parenting Scale assesses over-reactive and lax parenting. It is associated with home observations of discipline and child behavior problems, and with parent-reported child problem behavior [22]. Scores range from 1 to 7, with 1 representing the absence of over-reactivity or laxness and 7 representing extreme over-reactivity or laxness (Over-reactivity α = 0.49; Laxness α = 0.56).

Child Health Behaviors. The MacArthur Health Behavior Questionnaire (HBQ; [23]) is a parent-completed questionnaire measure of child behavior. We selected the Depression, Overanxious, Oppositional Defiant, Conduct Problem, Inattention, and Impulsivity subscales (36 items for ages 4–8; 50 items for ages 9–18). The Externalizing composite for the younger and older child versions respectively consisted of: Conduct (α = 0.60; n/a), Inattention (α = 0.79; α = 0.62), Impulsivity (α = 0.63; α = 0.60), and Oppositional Defiant (α = 0.60; α = 0.62); the Internalizing Composite for the younger and older child versions consisted of: Depression (n/a; α = 0.60), and Overanxious (α = 0.60; α = 0.78). Items were averaged for each subscale, and subscales were subsequently averaged to form the Externalizing and Internalizing composite score. The results from the younger and older child versions of the HBQ were standardized and combined to form an internalizing and externalizing composite score variable.

Adult Oral Health Behavior (AOHBM). The AOHBM is an 18-item questionnaire that assesses daily oral health maintenance (e.g., brushing/flossing), consumption of cariogenic snacks/drinks, tobacco use, history of tooth loss, and use of dental services.

Oral Health Socialization Measure (OHSM). OHSM is a 33-item parent-report questionnaire that assesses children’s daily oral health maintenance (e.g., brushing/flossing), parents’ supervision and reinforcement of child daily oral health maintenance, children’s consumption of cariogenic snacks/drinks, and children’s use of dental services.

Parent-Child Interaction Coding (PCI). PCI Coding of video recordings yielded a measure of observable warmth and responsiveness from parent to child. Two independent coders viewed and scored the parent-child interactions, achieving interrater reliability of Finn’s r = 0.94 and 0.89 for warmth and responsiveness, respectively, on 100% percent of videos. Warmth is captured by the parent’s words, tone, and body language, and the code ranges from 1 (cold) to 5 (very warm). Responsiveness refers to the attunement a parent shows his or her child on a moment to moment basis. This code ranges from 1 (very low responsiveness; parent focused on him or herself) to 4 (very high responsiveness; parent appears child-centered).

## 3. Results

### 3.1. Study 1: Results

The following constitutes the key findings of the focus groups.

Grenadian parents consistently reported personal finances and the economy as primary stressors. Parenting, supervision of children, work, stress, and a lack of community support were also commonly indicated. A lack of paternal support was mentioned frequently, as many of the mothers are single parents.

To help cope with stressors, parents said they rely on extended family, friends, school counselors, and the church. Some parents said they also work more to make more money (e.g., multiple jobs).

Parents reported handling child misbehavior by taking away privileges or possessions and discussing limits to behaviors. Additionally, parents employ scolding, corporal punishment, and time-outs. Parents said that they currently use physical punishment less than they have in the past and have increasingly turned to a non-physical discipline as corporal punishment becomes less accepted by society.

Parents said they tried to ensure their families’ health through nutrition, good hygiene, regular checkups, and exercise. Parents emphasized barriers to healthy nutrition for children, such as the popularity and inexpensiveness of snacks, such as chips. Furthermore, parents said that their children do not eat fruit they include in their lunch box, which is frustrating because fruit is expensive.

Participants commented that oral health is a major issue in their country, and they brush 2–3 times per day, floss occasionally, and monitor sweets. Parents report chewing on sugar cane to prevent cavities. Additionally, parents observed that dentists are intimidating, expensive, and unclear, and they tend to visit the dentists for treatment, not prevention. Participants stated that dentists at the free clinic in Grenada only perform extractions, and it often takes an entire day to be treated.

Parents reported a strong interest in programs to help families, such as information about tooth decay, anger management, better communication, saving money, and following a balanced diet. Parents said the best outlets to these programs are usually through health centers, TV, and local schools.

### 3.2. Study 2: Results

#### 3.2.1. Descriptive Statistics

See Table 2 for descriptive statistics. Participants reported somewhat unhappy relationships, with the mean just above the cut-score for clinical distress (normed in high-income countries). Parenting measures do not have norms, but the sample demonstrated substantial variability in discipline style, with a notable portion of the sample indicating very permissive or very over-reactive styles. Child psychological symptoms were, on average, in the normal range.

#### 3.2.2. Associations of Oral Health Behaviors and Other Variables

Parent and child oral health behaviors were significantly associated (see Table 3). Adult and child brushing frequency were positively correlated (*r* = 0.47, *p* < 0.001), as were adult and child brush time (*r* = 0.64, *p* < 0.001) and adult and child flossing frequency (*r* = 0.43, *p* = 0.002).

Consistent with hypotheses, relationship satisfaction was positively correlated with adult flossing behavior (*r* = 0.41, *p* = 0.021). Although not specifically hypothesized, child internalizing behavior was positively associated with both adult tooth loss to decay (*r* = 0.31, *p* = 0.025) and tooth loss to injury (*r* = 0.30, *p* = 0.029), both of which are consistent with the pattern of findings obtained in the U.S. sample about relations between family functioning and oral health [6]. Non-significant associations between family and oral health variables are listed in Table 3.

Inconsistent with hypotheses, we found that relationship satisfaction was negatively correlated with child brushing time, in that the more satisfaction participants reported, the less time they reported their children spend brushing their teeth (*r* = −0.41, *p* = 0.021). Also, inconsistent with hypotheses, both child externalizing and internalizing behaviors were positively correlated with adult brush time (*r* = 0.35, *p* = 0.011 and *r* = 0.32, *p* = 0.008 respectively). We ran a post-hoc regression model to test the association between relationship satisfaction and lax parenting in predicting child brushing behavior. This model was significant (adjusted R square = 0.42, *p* < 0.001). After controlling for relationship satisfaction, lax parenting still significantly predicted poor child brushing habits (Beta = −0.43; *t* = −3.10, *p* = 0.004).

#### 3.2.3. Motivational Interviewing Procedure and Results

Participants noted a range of dental issues, such as caries, extractions, and tooth decay. Most said their teeth were not in great overall health, and they did not often attend the dentist due to cost and inconvenience. All but one participant noted they only go to the dentist when they have dental pain. Some found dentists extremely helpful, but others found them fear-inducing.

Due to time constraints, only 13 participants were able to review each menu item. All expressed a desire and willingness to make sure their children brush their teeth at least twice a day and give their children a toothbrush for school. All expressed a desire to replace their children’s toothbrushes every four months, and the majority of participants expressed a desire to supervise children while they floss, bring their children to the dentist twice per year, and limit their children’s sugar intake. Yet, most parents felt powerless to limit sugar when their children are not always in their sight. Resistance to the supervision of flossing stemmed from the fact that some participants do not floss themselves. Finally, financial difficulty was the main reason participants gave for not taking their children to the dentist twice a year.

## 4. Discussion

### 4.1. Study 1: Discussion

The themes discussed in the focus groups suggested more similarities than differences between U.S. and Grenadian parents. Grenadian parents have a strong desire to instill healthy behaviors within their children and experience many of the same issues as low-income US parents. Grenadian families are often single-parent homes, which poses challenges to financial stability and parental supervision of children.

The differences lie mainly in oral health. Grenadian families are less likely to seek preventive dental care and have a more fatalistic view of oral health outcomes. Grenadians also report culture-specific health-promotion behaviors, such as chewing on sugar cane, which exposes the teeth to elevated sugar levels. This causes caries, but it also stimulates saliva and mechanically removes debris, which can help prevent caries. Generally, chewing on sugar cane slightly increases the risk of cavities, but only with a small effect size [24,25].

The similarities that emerged suggest that measures of family functioning and oral health might perform similarly in Grenadian families as they do in American samples [8], which suggests U.S. prevention approaches that leverage the relation between family functioning and child oral health might be applicable to Grenadian families. Therefore, Study 2 was conducted to obtain quantitative information about Grenadian oral health behaviors and to assess whether the measures and prevention approaches from American studies would function as expected.

### 4.2. Study 2: Discussion

Parent and child oral health behaviors were significantly associated with one another, suggesting that parents and children perform similar oral hygiene routines. It should be noted that parental reports of brushing length are surprisingly high. Whereas, American parents report that children brush 1.78 times per day (*SD* = 0.53) and for 1.61 min (*SD* = 1.0), Grenadian parents are reporting 1.51 times per day (*SD* = 0.61) but for 2.33 min (*SD* = 1.05). It is possible that this reflects a true difference in brushing behaviors or that social desirability influenced the validity of these data. Additionally, research shows a significant difference between the length of time individuals believe they brush and actual time spent brushing (more than 134 s versus less than 84 s, respectively) [26].

A majority of participants were single parents, and those in relationships reported fairly low relationship satisfaction. Child externalizing and internalizing behaviors were significantly positively associated, indicating an expected, more general, construct of child problems. Finally, as expected, those who were more satisfied in their adult relationships reported fewer problems with their children.

There were indications that oral health and family variables were associated in ways consistent with emerging findings with American samples [8]. Those with higher relationship satisfaction endorsed longer adult flossing, and child internalizing problems were associated with adult tooth loss to decay and injury. Although there was no significant association between over-reactive discipline and child brushing frequency, the effect size (*r* = −0.26) is what we might expect. This is consistent with previous findings that high-conflict family environments can be linked to poorer oral health outcomes, and victims of physical or emotional aggression had higher adult caries [6]. Having greater relationship stress or child behavior problems is a stressor to the family and likely takes up family resources, leading to worse oral health maintenance. However, there were some unexpected findings. Child internalizing and externalizing behavior was positively associated with adult oral health, and adult relationship satisfaction was negatively associated with child brushing time. It is not clear whether these unexpected findings are attributable to measurement error (e.g., response biases), culturally specific properties of the measures, or whether the constructs themselves are differently related across the two cultural contexts. Furthermore, because this is not a clinical sample, the rates of internalizing and externalizing problems are relatively low.

Over-reactive parenting was common, suggesting harsh discipline continues to be normative among Grenadian families. Parenting style was not associated with relationship satisfaction, child problems, or any oral health measures, and the parenting subscales had poor reliability estimates in this sample. It is possible that these scales are not a good fit for the Grenadian parents, and that more culturally appropriate parenting measures need to be developed. These findings might also indicate that because harsh parenting is culturally normative, it is less systematically impactful on child outcomes. A multi-method approach would help bring clarity.

The observational coding of parent-child interactions revealed no significant associations with self-reported parenting behaviors, or with other relationship or oral health behaviors. Observations of warmth indicated mostly neutral interactions between parents and children, with moderate parental responsiveness. These averages are lower than we have observed in American samples; it may be that we were less successful in evoking naturalistic, generalizable interactions in this analog situation, or that coding warmth and responsiveness in Grenadian families should be adjusted to account for cultural differences in expressiveness.

Oral hygiene, relationship satisfaction, and child behavior measures seem to have functioned well in this sample, showing associations among family stressors and oral health. Further development of culturally sensitive parenting self-report measures and parent-child observation protocols would help identify specific family constructs that could be targeted in oral-health behavior interventions. The openness and interest regarding oral health during the motivational interviews suggests that further development of family-friendly behavioral health interventions would be well-received.

Limitations of this study should be noted. Both studies were convenience samples that might limit generalizability of the findings. Furthermore, the sample size for Study 2, although typical for observational studies, does not provide power only to detect small effect sizes. The motivational interviewing portion of Study 2, given its modest size, should be interpreted as a pilot study.

## 5. Conclusions

Oral hygiene, relationship satisfaction, and child behavior measures seem to have functioned well in this sample and demonstrated the association among family stressors and oral health. Further development of parenting self-report measures and structured parent-child interaction assessment protocols that are culturally sensitive would help identify specific constructs that could be targeted in health behavior interventions within a family context. The openness and interest regarding oral health during the motivational interviews suggests that further development of family-friendly behavioral health interventions would be well received, as would school and community policies that would support parents’ desire to reduce children’s access and exposure to sugary foods and drinks.

## Figures and Tables

**Table 1:** Study 2 Demographics.

Demographic Varable	Women (*n* = 48)	Men (*n* = 6)	Children (*n* = 54)
Age * (*M* (*SD*))	35.23 (7.43)	40.39 (9.77)	8.55 (2.66)
Employment * (n, %)			
Full-time	21 (42%)	22 (59.5%)	
Part-time	10 (20%)	9 (24.3%)	
Homemaker	9 (18%)	0 (0%)	
Unemployed	6 (12%)	2 (5.4%)	
Disabled	1 (2%)	0 (0%)	
Missing	3 (6%)	4 (10.8%)	
Education * (n, %)			
Some High School	20 (40%)	18 (48.6%)	
High School Diploma or GED	13 (26%)	9 (24.3%)	
Some College	8 (16%)	4 (10.8%)	
College Graduate	6 (12%)	2 (5.4%)	
Missing	3 (6%)	4 (10.8%)	
Marital Status (n, %)			
Single	17 (31.5%)		
Married	11 (20.4%)		
In a Relationship	5 (9.3%)		
Living with Partner	17 (31.5%)		
Missing	4 (7.4%)		

* Participants reported on both their own and their partner’s demographic information.

**Table 2:** Descriptive Statistics for Study 2 Variables.

Self-Reported Variables	Mean	*SD*	Range	Possible Range	Clinical Cut-Score
Oral Health					
Adult Brushing Frequency (times per day)	1.35	0.52	0–2	0–4	–
Adult Brushing Time (in minutes)	2.65	1.28	1–5	1–6	–
Adult Flossing Frequency (times per week)	1.73	1.51	0–4	0–4	–
Adult Tooth Loss—Decay (number of teeth)	1.02	1.73	0–4	0–5	–
Child Brushing Frequency (times per day)	1.51	0.61	0–3	0–4	–
Child Brushing Time (in minutes)	2.33	1.05	1–5	1–6	–
Child Flossing Frequency (times per week)	1.15	1.23	0–4	0–4	–
Child Tooth Loss—Decay (number of teeth)	0.67	1.26	0–4	0–5	–
Family Functioning					
Relationship Satisfaction (higher = more satisfied)	13.72	4.07	7.00–21.00	0–21	13.5
Lax Discipline (higher = more lax)	2.97	1.15	1.17–6.17	1–7	N/A
Overreactive Discipline (higher = more overreactive)	3.49	1.20	1.00–5.80	1–7	N/A
Child Internalizing Problems—Younger Children (4–8)	0.30	0.30	0.00–1.13	0–2	0.72
Child Internalizing Problems—Older Children (9–18)	0.61	0.31	0.05–1.18	0–2	0.72
Child Externalizing Problems—Younger Children (4–8)	0.49	0.32	0.00–1.06	0–2	0.68
Child Externalizing Problems—Older Children (9–18)	0.61	0.33	0.07–1.53	0–2	0.68
Observed parental warmth	3.11	0.77	1.00–5.00	1–5	–
Observed parental responsiveness	2.68	0.76	1.00–4.00	1–5	–

**Table 3:** Correlations Among Study 2 Variables.

Self-Reported Variables	Child Ext.	Child Int.	Over-Reactive Discipline	Lax Discipline	Observed Parental Warmth	Observed Parental Responsiveness	Adult Brush Freq	Adult Brush Time	Adult Floss Freq	Adult Lost Tooth Decay	Adult Lost Tooth Injury	Child Brush Freq	Child Brush Time	Child Floss Freq	Child Lost Tooth Decay	Child Lost Tooth Injury
Relationship Satisfaction	−0.48 **	−0.45 **	−0.22	−0.15	−0.23	−0.08	−0.03	−0.32	0.41 *	−0.02	0.00	−0.10	−0.41 *	−0.14	−0.18	−0.06
Child Ext.	−	0.48 **	0.15	−0.01	0.32 *	0.15	−0.03	0.35 *	−0.05	−0.04	0.20	−0.01	0.17	−0.19	−0.05	0.19
Child Int.		–	0.12	−0.13	0.07	−0.07	0.20	0.32 *	−0.08	0.31 *	0.30	0.06	0.20	−0.12	0.27	0.23
Overreactive Discipline			–	0.17	0.06	−0.01	−0.14	0.20	−0.14	0.04	0.07	−0.26	0.02	−0.05	0.26	0.23
Lax Discipline				–	−0.06	−0.08	0.04	0.06	−0.08	−0.05	−0.14	−0.14	−0.06	0.00	0.02	0.04
Observed parental warmth					–	0.80 **	−0.03	0.21	0.15	0.15	0.06	0.03	−0.05	0.12	−0.06	0.08
Observed parental responsiveness						–	−0.19	0.03	0.17	0.11	−0.07	−0.17	−0.03	−0.01	−0.10	0.00
Adult Brush Freq							–	0.02	−0.03	−0.03	0.00	0.47 **	−0.08	0.15	0.16	−0.25
Adult Brush Time								–	0.14	0.10	0.12	−0.08	0.64 **	−0.03	0.16	0.20
Adult Floss Freq									–	0.10	0.21	−0.00	−0.03	0.43 **	0.10	0.22
Adult Lost Tooth Decay										–	−0.08	−0.05	0.07	0.04	0.25	0.03
Adult Lost Tooth Injury											–	−0.03	0.12	0.11	0.18	0.19
Child Brush Freq												–	-0.02	0.02	−0.01	−0.21
Child Brush Time													–	−0.06	-0.01	−0.06
Child Floss Frequency														–	0.09	0.08
Child Lost Tooth Decay															–	0.18

Note: * *p* < 0.05; ** *p* < 0.01.

## Appendix A

Questions in the structured interview guide.

Family:

1. What are the stressors that are on your family?
2. What are the things that help you cope with those stressors?
3. All children misbehave. How do you handle child misbehavior?
4. All couples have disagreements. How do you handle disagreements with your partner?

Health:

5. What kinds of things do you do to keep your family healthy?
6. Tell us what you think about teeth and cavities.
7. What sorts of things do you do or your kids do to prevent cavities? What helps make that easier or harder?
8. What sorts of snacks and drinks do your kids have? What makes it easier or harder to have them make the choices you would like them to?

Dentists:

9. Tell me the first words or images that come to your mind when you hear the word “dentist”.
10. What are your family’s attitudes and beliefs about dentists and care of your mouth?
11. What makes it difficult for you to see a dentist?
12. Tell me about your last experience seeing a dentist.
13. Tell me what factors contribute to you following or not following a dentist’s recommendations (like changing your diet or getting cavities filled).

Other:

14. Would you ever be in a program for families to help the parents support their children’s healthy development? What if it were at the school? What if it were just for you and your spouse? What if you could do it on your own time?
